# Comparison of Suture-Based and Collagen-Based Vascular Closure Devices for Large Bore Arteriotomies—A Meta-Analysis of Bleeding and Vascular Outcomes

**DOI:** 10.3390/jcdd9100331

**Published:** 2022-09-30

**Authors:** Sumit Sohal, Sheetal Vasundara Mathai, Sanjana Nagraj, Krishna Kurpad, Kandarp Suthar, Harsh Mehta, Komaldeep Kaur, Najam Wasty, Sergio Waxman, Marc Cohen, Gautam K. Visveswaran, Rajiv Tayal

**Affiliations:** 1 Division of Cardiology, Department of Medicine, RWJ-BH Newark Beth Israel Medical Center, 201 Lyons Ave, Newark, NJ 07112, USA; 2 Department of Medicine, Jacobi Medical Center, 1400 Pelham Parkway South, New York, NY 10461, USA; 3 Department of Medicine, RWJ-BH Saint Barnabas Medical Center, 94 Old Short Hills Rd, Livingston, NJ 07039, USA; 4 Department of Medicine, Rutgers New Jersey Medical School, 150 Bergen Street, Newark, NJ 07101, USA; 5 Division of Cardiology, Department of Medicine, University of Kansas Medical Centre, 3901 Rainbow Boulevard, Kansas City, KS 66160, USA; 6 Department of Medicine, Crozer Chester Medical Center, 1 Medical Center Blvd, Upland, PA 19013, USA; 7 Division of Cardiology, Department of Medicine, Valley Health System, 1200 East Ridgewood Avenue, suite 301, Ridgewood, NJ 07450, USA

**Keywords:** vascular closure devices, suture, collagen, transcatheter aortic valve replacement, bleeding outcomes, vascular outcomes

## Abstract

Background: Large bore access procedures rely on vascular closure devices to minimize access site complications. Suture-based vascular closure devices (S-VCD) such as ProGlide and ProStar XL have been readily used, but recently, newer generation collagen-based vascular closure devices (C-VCD) such as MANTA have been introduced. Data on comparisons of these devices are limited. Methods: PubMed, Scopus and Cochrane were searched for articles on vascular closure devices using keywords, (“Vascular closure devices” OR “MANTA” OR “ProStar XL” OR “ProGlide”) AND (“outcomes”) that resulted in a total of 875 studies. Studies were included if bleeding or vascular complications as defined by Valve Academic Research Consortium-2 were compared between the two types of VCDs. The event level data were pooled across trials to calculate the Odds Ratio (OR) with 95% CI, and analysis was done with Review Manager 5.4 using random effects model. Results: Pooled analyses from these nine studies resulted in a total of 3410 patients, out of which 2855 were available for analysis. A total of 1229 received C-VCD and 1626 received S- VCD. Among the patients who received C-VCD, the bleeding complications (major and minor) were similar to patients who received S-VCD ((OR: 0.70 (0.35–1.39), *p* = 0.31, I^2^ = 55%), OR: 0.92 (0.53–1.61), *p* = 0.77, I^2^ = 65%)). The vascular complications (major and minor) in patients who received C-VCD were also similar to patients who received S-VCD ((OR: 1.01 (0.48–2.12), *p* = 0.98, I^2^ = 52%), (OR: 0.90 (0.62–1.30), *p* = 0.56, I^2^ = 35%)). Conclusions: Bleeding and vascular complications after large bore arteriotomy closure with collagen-based vascular closure devices are similar to suture-based vascular closure devices.

## 1. Introduction

The last decade witnessed a rapid increase in the number of percutaneous catheter-based procedures including but not limited to transcatheter aortic valve replacement (TAVR), insertion of large bore mechanical circulatory support devices (MCS), mitral or tricuspid clipping, percutaneous endoscopic abdominal aortic aneurysm repair, and thoracic endovascular aortic repair [1,2,3]. To ensure the minimally invasive nature of these procedures, large bore access (LBA) is required but may be associated with access site complications including vascular and bleeding complications which lead to increased costs, morbidity, mortality, and length of stay [4,5]. Deployment of preventive strategies with the use of vascular closure devices (VCDs) play an important role in management of patients with LBA but their failure remain one of the reason for these complications [6,7]. Suture-based vascular closure devices (S-VCD) such as Perclose ProGlide and ProStar XL (Abbott Vascular, Santa Clara, CA, USA) have been readily used for closure of LBA, however LBA dedicated collagen-based vascular closure devices (C-VCD) (MANTA; Teleflex, Wayne, PA, USA) have been introduced into the market in a bid to decrease access site complications [8].

Suture-based VCDs work on the principle of a pre-tied slip knot which is percutaneously delivered at the site of arteriotomy to close the access site [9,10] whereas collagen-based VCDs (here MANTA^R^) consist of a hemostatic plug (collagen) on the outside of the artery which is held in place by a suture linked to a small molded polymer toggle positioned inside the artery. A small stainless steel lock is used to secure the components in a sandwich and on either side of the arteriotomy site [11]. Several published observational studies compare the outcomes among S-VCD and C-VCD [12,13,14,15,16,17,18], however the recent MASH and CHOICE-CLOSURE trials are the only randomized controlled trials that compare the two VCDs [19,20]. We aim to pool data to present a large, comprehensive, and updated meta-analysis to compare the bleeding and vascular complications of S-VCDs and C-VCDs in LBA procedures. To our knowledge, this is the most up-to-date meta-analysis to compare outcomes among the suture-based and collagen-based vascular closure devices.

## 2. Methods

### 2.1. Data Sources and Search Strategy

**Figure 1:**
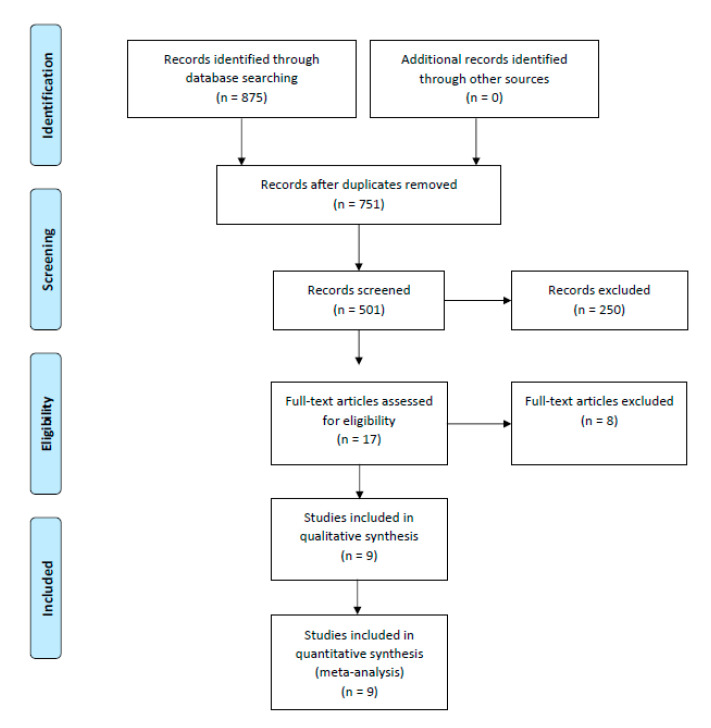
PRISMA flow diagram for systematic literature review process.

We searched PUBMED, SCOPUS and COCHRANE for eligible studies from inception to 31 January 2022. The keywords used were (“Vascular closure devices” OR “MANTA” OR “ProStar XL” OR “ProGlide”) AND (“outcomes”). Studies were included if bleeding or vascular complications as defined by Valve Academic Research Consortium-2 (VARC-2) [21] were compared between the two types of VCDs. Guidelines reported in Preferred Reporting Items for Systematic Reviews and Meta-Analysis (PRISMA) were used to conduct and report this meta-analysis [22] (Figure 1).

### 2.2. Data Extraction and Outcomes

Studies retrieved from the databases were reviewed by authors SS and SK independently and after removal of all duplicates, articles were screened by their title and abstract followed by full text level review. Articles were selected for inclusion if they met the aforementioned eligibility criteria, and any discrepancies were resolved by discussion and mutual consensus. Quality assessment of the included studies was done using ROBIN-I tool for non-randomized studies and ROB-2 tool for randomized studies (Supplementary Files) [23,24]. The primary outcomes of interest were bleeding and vascular outcomes which were further divided into major and minor outcomes. The secondary outcomes include vascular closure device failure, rates of 30-day all-cause mortality and stroke. These outcomes in the included studies were defined by VARC-2 criteria [21]. A secondary analysis of similar outcomes was also performed to compare the two types of VCDs without inclusion of studies using ProStar XL S-VCD.

### 2.3. Statistical Analysis

Review Manager 5.4 (Review Manager (RevMan) [Computer program]. Version 5.4, The Cochrane Collaboration, 2020) was used for statistical analysis. Event level data wasc extracted from studies for each arm and used to calculate the odds ratio (OR) with 95% confidence intervals (CI). Random effects model was selected to account for heterogeneity among different studies. Subgroup differences were tested using χ^2^ test and *p* value ≤ 0.05 was considered statistically significant. Heterogeneity was assessed using Higgins and Thompsons’ I^2^ [25]. I^2^ of 25% represent low heterogeneity, 50% represent moderate heterogeneity, and 75% represent high heterogeneity.

## 3. Results

**Figure 2:**
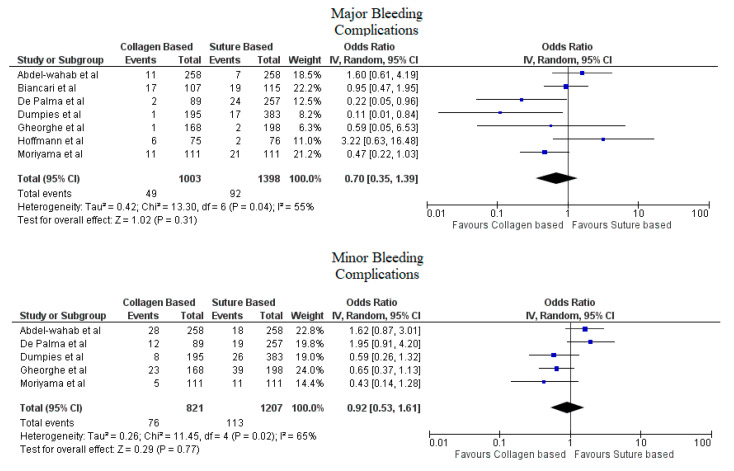
Forest plot for comparison of major and minor bleeding complications between collagen vs. suture-based vascular closure devices for large bore arteriotomies, CI: confidence interval, IV: Instrumental variable.

**Figure 3:**
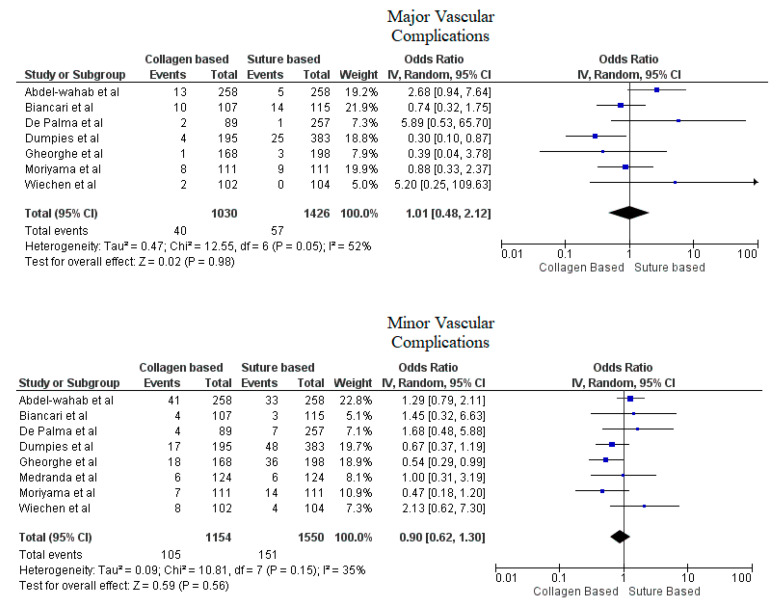
Forest plot for comparison of major and minor vascular complications between collagen vs. suture-based vascular closure devices for large bore arteriotomies, CI: confidence interval, IV: Instrumental variable.

**Figure 4:**
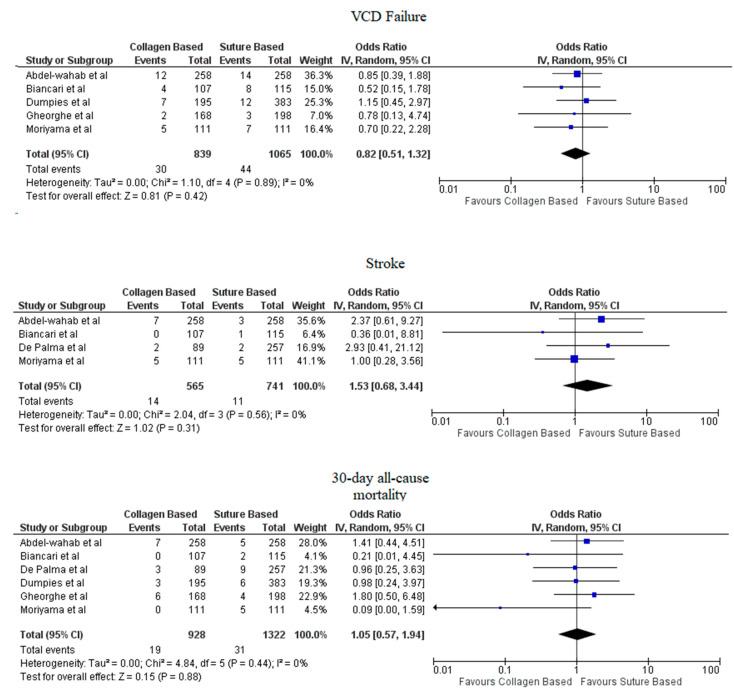
Forest plot of procedural and post-procedural complications of collagen-based vs. suture vascular closure device for large bore arteriotomies. CI: Confidence interval, IV: Instrumental variable, VCD: Vascular closure device.

Pooled analyses from these nine studies resulted in a total of 3410 patients. Study characteristics are described in Table 1. The final analysis included 2855 patients. C-VCDs were used in 1229 (43.05%) patients whereas 1626 (56.95%) received a suture-based device. Among the patients who received C-VCD, major bleeding complications occurred in 49 out of 1003 patients whereas in S-VCD group, it occurred in 92 out of 1398 patients (4.89% versus 6.58%, OR: 0.70 (0.35–1.39), *p* = 0.31, I^2^ = 55%)). The major vascular complications occurred in 40 out of 1030 patients who received C-VCD whereas complications occurred in 57 out of 1426 patients in the S-VCD group (3.88% versus 3.99%, (OR: 1.01 (0.48–2.12), *p* = 0.98, I^2^ = 52%)). There were 76 patients in the C-VCD group of 821 patients who had minor bleeding complications as compared to 113 patients in the S-VCD group of 1207 patients (9.26% versus 9.36%, (OR:0.92 (0.53–1.61), *p* = 0.77, I^2^ = 65%)). Similarly, in C-VCD group 105 patients had minor vascular complications out of 1154 patients whereas in S-VCD group, 151 out of 1550 patients had this type of complication (9.10% versus 9.74%, (OR:0.90 (0.62–1.30), *p* = 0.56, I^2^ = 35%)). The comparison of major and minor bleeding as well as vascular outcomes are represented in Figure 2 and Figure 3. To account for significant heterogeneity in the bleeding and vascular complications, analysis was adjusted to make the two groups less heterogeneous as possible, but the outcomes did not differ significantly (Supplemental Figure S1). The rates of VCD failure, 30-day all-cause mortality and stroke are represented in Figure 4. The comparison of primary and secondary outcomes of secondary analysis without Prostar XL is shown in Supplementary Figures S2–S4.

## 4. Discussion

Vascular and bleeding complications remain the most common adverse outcomes associated with procedures like TAVR, which use large bore access. In an analysis of 34,893 patients undergoing TAVR, 9.3% of the patients experienced a vascular complication (major or minor) whereas 7.6% had an in-hospital bleeding event [26]. Although over the last several years rates of bleeding and vascular complications have significantly decreased, they still remain a matter of concern. These complications not only lead to longer hospital stays but are also associated with higher 30-day mortality and rehospitalizations [26,27,28]. The VCDs have played a significant role in reducing risk of vascular complications but failure of their deployment remains a genuine concern. In a patient level meta-analysis of 891 patients, 3.1% of the complications occurred due to incomplete arteriotomy closure, which constituted about 34% of the total vascular complications [29]. Meighem et al., in their study of 986 patients, reported closure device failure as a cause of 64% of the major vascular complications and 29% of life threatening/disabling bleeding [6]. Failure of vascular closure devices is an independent predictor of vascular complications [28], and further, lead to higher 30-day rates of major bleeding and transfusions [30]. Among the devices available, suture-based VCDs have been most commonly used for access closure but in an attempt to improve rates of complications, MANTA, a collagen-based VCD was introduced as a dedicated large bore access closure device. The SAFE MANTA trial demonstrated the safety and efficacy of the MANTA percutaneous closure device in a large single arm prospective multicenter investigation [11]. After establishment of its safety and efficacy profile, several retrospective studies and randomized controlled trials have been done to compare the efficacy of this C-VCD with that of S-VCD but most of them are in a small number of patients. In an attempt to compare these VCDs in a pooled analysis in our study, we found that use of either S- or C-VCDs for LBA resulted in similar rates of both major and minor bleeding and vascular complications. The rates of VCD failure do not differ between both groups, and the rates of 30-day all-cause mortality and stroke rates are similar in both groups. To the best of our knowledge this is the largest study directly comparing the two VCDs.

Among the two commonly used suture-based VCDs, ProGlide has been shown to have better efficacy than Prostar XL. A multicenter prospective study of 2583 patients addressing the procedural, 30 days, and a one-year comparative performance of the ProGlide versus Prostar VCDs undergoing TAVR showed a significantly greater reduction of the composite endpoint of cardiovascular mortality, bleeding and vascular complications at 30 days (aOR: 0.80 (95%CI 0.65–0.99); *p* = 0.043), a higher procedural success (99.2% versus 97.5%, *p* = 0.001) with ProGlide as compared to Prostar XL and no significant difference in the primary end point at one-year follow up (aHR 0.88 (95%CI: 0.72–1.10) *p* = 0.902) [31]. Several other studies have confirmed better performance of ProGlide as compared to Prostar XL [32,33]. To account for the superiority of ProGlide VCD, we did a modified analysis with exclusion of studies using Prostar XL as the lone suture-based VCD. The findings remained similar despite removal of studies which used ProStar XL as the suture-based vascular closure device. There was no significant difference in bleeding outcomes ((Major bleeding: OR: 0.85 (0.39–1.83), *p* = 0.67, I^2^ = 61%), (Minor bleeding: OR: 0.80 (0.35–1.86), *p* = 0.61, I^2^ = 68%)) between C-VCD when compared to S-VCD (ProGlide only). Similarly vascular outcomes also did not differ significantly between C-VCD and S-VCD (ProGlide only) ((Major vascular: OR: 0.95 (0.42–2.12), *p* = 0.89 I^2^ = 59%), (Minor vascular: 0.96 (0.64–1.44), *p* = 0.84, I^2^ = 28%)) (Supplementary Figures S2 and S3). The secondary outcomes were also similar in both groups (Supplementary Figure S4).

The reduction of vascular and bleeding complications over the last decade are commendable and VCDs have played an important role in addition to measures such as reduction of sheath and device sizes, use of ultrasound guidance, and micropuncture access [18,26]. The newer C-VCD were introduced in hope of further reducing these complications, however they have failed to show superiority in the two major randomized controlled trials to prevent these outcomes [19,20]. Despite significant differences in the design and functioning of the two devices, they have maintained very similar rates of complications. These findings highlight the role of intrinsic patient or access site factors that may lead to similarity of the outcomes with these two devices. The striking similitude of predictors of vascular outcomes with use of either of these VCDs corroborate the theory of these factors. Variables such as age [34,35], female gender [36], severity of calcification or peripheral vascular disease [10,29,36], increased sheath size [10], higher sheath to femoral artery ratio [35,37,38], depth of arteriotomy site, and femoral artery size [10,29] play an important role in speculating vascular outcomes and have been shown as predicting variables for vascular outcomes with both VCDs. Calcification in the artery may lead to failure of VCD deployment despite having different mechanisms of failure with each device. Where in S-VCD, failure can be due to suture tear or incomplete apposition of highly calcified walls, toggle-plug malapposition due to calcium can lead to failure of C-VCD [19]. On the similar terms, both categories of devices may lead to other complications including distal embolization of the plug (in C-VCD) or foot plate (in S-VCD) and can also lead to infections of the arterial or access site [39,40,41]. Some of these factors may be non-modifiable, such as age and gender, but the introduction of intravascular lithotripsy facilitated transfemoral TAVR [42,43] or the reduction in sheath sizes with newer devices [44] may play important roles in the reduction of poor outcomes in LBA and further delineate any differences in the outcomes of these two VCDs.

Currently, with no major differences, the onus of selecting the devices now lies on individual operators, patient characteristics, and institutional preference. Studies have described steeper learning curves for MANTA [45], defined it as an “easy to use” device while significantly decreasing the time to hemostasis [19,20] but its effect on major outcomes have not been demonstrated [12]. Furthermore, along with similar outcomes, most of the studies have demonstrated similar lengths of stay for patients undergoing either kind of device [17,18,19,20]. With no demonstrable difference in outcomes or an economic dividend, choosing an expensive single device like MANTA over available cheaper devices may be a bottleneck in the widespread use of this device.

## 5. Limitations

There were some limitations in our study. Efforts to group similar populations were made, however there was diversity in the baseline inclusion criteria of the patients which may impact the results. Event level data was used for analysis and time to event analysis was not done as the hazard ratio was not readily available for each outcome in the studies. Finally, though a random effects model was used to account for heterogeneity, it does not rule out the minute differences in various trials including, but not limited to, different classes of types of devices, sheath outer diameter, differences in baseline characteristics like background therapy including antiplatelet therapy or anticoagulation strategies, vessel characteristics such as calcification, social characteristics, and the difference in the definition of similar-sounding outcomes or different outcomes.

## 6. Conclusions

An analysis of the seven observational and two randomized controlled trials indicates that the vascular and bleeding complications after large bore arteriotomy closure with collagen-based vascular closure devices are similar to suture-based vascular closure devices. No difference was found in the rate of VCD failure, 30-day all-cause mortality and stroke between the two groups. These results are hypothesis-generating and further large randomized controlled trials are required to compare these devices and study the cost-effectiveness of collagen-based VCDs over suture-based VCDs.

## Figures and Tables

**Table 1:** Summary of studies included for meta-analysis and brief description of study outcomes.

S. No	Study	Type of Study	Year	Comparison	Primary Outcome
1.	De Palma et al. [15].	Observational	2018	MANTA vs. Prostar XL	Closure success and time to hemostasis
2.	Biancari et al. [12].	Observational	2018	MANTA vs. ProGlide	Invasive treatment of bleeding, life-threatening/disabling bleeding and major vascular complications
3.	Hoffman et al. [13].	Observational	2018	MANTA vs. ProGlide	Vascular complications or non-planned vascular surgery
4.	Gheorghe et al. [14].	Observational	2019	MANTA vs. Prostar XL	Acute closure success and occurrence of any access site related vascular injury as well as major and life threatening/disabling bleeding
6.	Moriyama et al. [16].	Observational	2019	MANTA vs. ProGlide	Bleeding and vascular complications
7.	Wiechen et al. [19].	Randomized Controlled Trial	2021	MANTA vs. ProGlide	Composite end point of access site related major and minor vascular complications
8.	Medranda et al. [18].	Observational	2021	MANTA vs. ProGlide	Vascular closure device success
9.	Dumpies et al. [17].	Observational	2021	MANTA vs. ProGlide	In-hospital vascular and access-site-related complications
10.	Abdel-Wahab et al. [20].	Randomized controlled trial	2021	MANTA vs. ProGlide	In-hospital access-site or access related major and minor vascular complications

## Data Availability

Studies used in this meta-analysis have been cited in this paper and are available publicly for analysis.

## Supplementary Materials

The following supporting information can be downloaded at: https://www.mdpi.com/article/10.3390/jcdd9100331/s1, Figure S1: Forest plot for analysis of major and minor bleeding complications (adjusted to decrease heterogeneity), CI: confidence interval, IV: Instrumental variable Figure S2: Forest plot for secondary analysis of major and minor bleeding complications (excluding Prostar XL S-VCD), CI: confidence interval, IV: Instrumental variable. Figure S3: Forest plot for secondary analysis of major and minor vascular complications (excluding Prostar XL S-VCD), CI: confidence interval, IV: Instrumental variable. Figure S4: Forest plot of for secondary analysis of secondary outcomes (excluding Prostar XL S-VCD): CI: Confidence interval, IV: Instrumental variable, VCD: Vascular closure device. Table S1: Assignment of variables to the outcomes. Table S2: Risk of bias analysis for Medranda et al. for primary and secondary outcomes. Table S3: Risk of bias analysis for Dumpies et al. for primary and secondary outcomes. Table S4: Risk of bias analysis for Noriaki et al. for primary and secondary outcomes. Table S5: Risk of bias analysis for Gheroge et al. for primary and secondary outcomes. Table S6: Risk of bias analysis for Hoffman et al. for primary and secondary outcomes. Table S7: Risk of bias analysis for Biancari et al. for primary and secondary outcomes. Table S8: Risk of bias analysis for DePalma et al. for primary and secondary outcomes. Table S9: Risk of bias analysis for Weichen et al. for primary and secondary outcomes. Table S10: Risk of bias analysis for Abdel-Wahab et al. for primary and secondary outcomes.
