# Supplementary material for: Comparison of Suture-Based and Collagen-Based Vascular Closure Devices for Large Bore Arteriotomies—A Meta-Analysis of Bleeding and Vascular Outcomes
Source: J Cardiovasc Dev Dis. 2022 Sep 30;9(10):331. doi: 10.3390/jcdd9100331 (PMC9604251; doi:10.3390/jcdd9100331)
Supplement: Supplementary file 1 [file jcdd-09-00331-s001.zip › jcdd-1844374-supplementary.pdf]

Supplementary Files

Major Bleeding Complications

Study or Subgroup	Collagen Based Events	Collagen Based Total	Suture Based Events	Suture Based Total	Weight	Odds Ratio IV, Random, 95% CI
Abdel-wahab et al	11	258	7	258	20.4%	1.60 [0.61, 4.19]
Biancari et al	17	107	19	115	25.7%	0.95 [0.47, 1.95]
De Palma et al	2	89	24	257	12.8%	0.22 [0.05, 0.96]
Dumpies et al	1	195	17	383	0.0%	0.11 [0.01, 0.84]
Gheorghe et al	1	168	2	198	6.0%	0.59 [0.05, 6.53]
Hoffmann et al	6	75	2	76	11.0%	3.22 [0.63, 16.48]
Moriyama et al	11	111	21	111	24.1%	0.47 [0.22, 1.03]
**Total (95% CI)**		**808**		**1015**	**100.0%**	**0.82 [0.43, 1.57]**
Total events	48		75			

Heterogeneity: Tau² = 0.29; Chi² = 9.73, df = 5 (P = 0.08); I² = 49%
Test for overall effect: Z = 0.59 (P = 0.56)

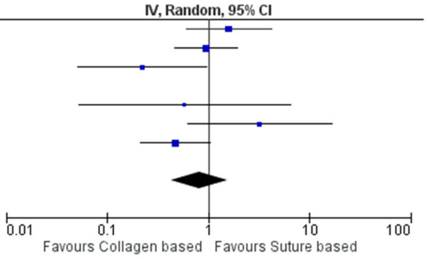

Minor Bleeding Complications

Study or Subgroup	Collagen Based Events	Collagen Based Total	Suture Based Events	Suture Based Total	Weight	Odds Ratio IV, Random, 95% CI
Abdel-wahab et al	28	258	18	258	28.9%	1.62 [0.87, 3.01]
De Palma et al	12	89	19	257	0.0%	1.95 [0.91, 4.20]
Dumpies et al	8	195	26	383	23.3%	0.59 [0.26, 1.32]
Gheorghe et al	23	168	39	198	30.7%	0.65 [0.37, 1.13]
Moriyama et al	5	111	11	111	17.0%	0.43 [0.14, 1.28]
**Total (95% CI)**		**732**		**950**	**100.0%**	**0.77 [0.43, 1.37]**
Total events	64		94			

Heterogeneity: Tau² = 0.20; Chi² = 7.36, df = 3 (P = 0.06); I² = 59%
Test for overall effect: Z = 0.89 (P = 0.37)

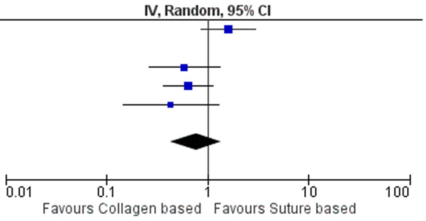

Major Vascular Complications

Study or Subgroup	Collagen based Events	Collagen based Total	Suture based Events	Suture based Total	Weight	Odds Ratio IV, Random, 95% CI
Abdel-wahab et al	13	258	5	258	20.8%	2.68 [0.94, 7.64]
Biancari et al	10	107	14	115	24.1%	0.74 [0.32, 1.75]
De Palma et al	2	89	1	257	0.0%	5.89 [0.53, 65.70]
Dumpies et al	4	195	25	383	20.4%	0.30 [0.10, 0.87]
Gheorghe et al	1	168	3	198	8.1%	0.39 [0.04, 3.78]
Moriyama et al	8	111	9	111	21.7%	0.88 [0.33, 2.37]
Wiechen et al	2	102	0	104	5.0%	5.20 [0.25, 109.63]
**Total (95% CI)**		**941**		**1169**	**100.0%**	**0.88 [0.42, 1.82]**
Total events	38		56			

Heterogeneity: Tau² = 0.39; Chi² = 10.19, df = 5 (P = 0.07); I² = 51%
Test for overall effect: Z = 0.36 (P = 0.72)

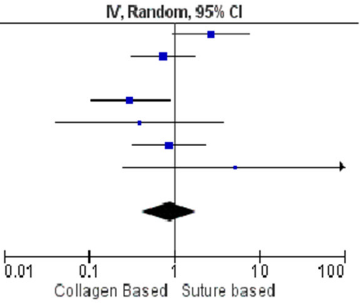

Supplementary Figure S1. Forest plot for analysis of major and minor bleeding complications (adjusted to decrease heterogeneity), CI: confidence interval, IV: Instrumental variable.

Major Bleeding Complications

Study or Subgroup	Collagen Based Events	Collagen Based Total	Suture Based Events	Suture Based Total	Weight	Odds Ratio IV, Random, 95% CI
Abdel-wahab et al	11	258	7	258	22.8%	1.60 [0.61, 4.19]
Biancari et al	17	107	19	115	27.2%	0.95 [0.47, 1.95]
Dumpies et al	1	195	17	383	10.3%	0.11 [0.01, 0.84]
Hoffmann et al	6	75	2	76	13.7%	3.22 [0.63, 16.48]
Moriyama et al	11	111	21	111	26.0%	0.47 [0.22, 1.03]
**Total (95% CI)**		**746**		**943**	**100.0%**	**0.85 [0.39, 1.83]**
Total events	46		66			

Heterogeneity: Tau² = 0.44; Chi² = 10.36, df = 4 (P = 0.03); I² = 61%
Test for overall effect: Z = 0.43 (P = 0.67)

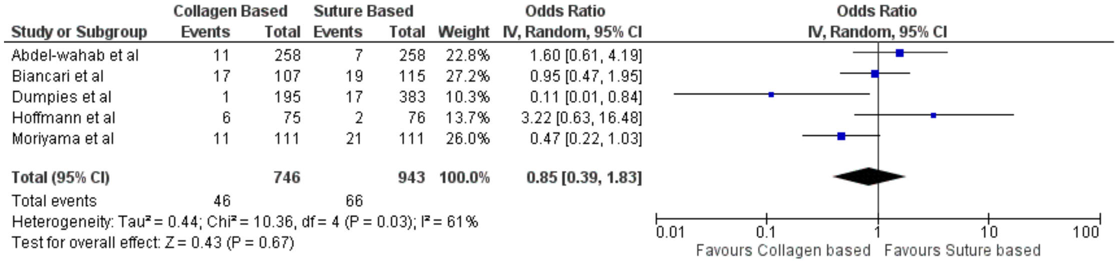

Minor Bleeding Complications

Study or Subgroup	Collagen Based Events	Collagen Based Total	Suture Based Events	Suture Based Total	Weight	Odds Ratio IV, Random, 95% CI
Abdel-wahab et al	28	258	18	258	39.1%	1.62 [0.87, 3.01]
Dumpies et al	8	195	26	383	33.9%	0.59 [0.26, 1.32]
Moriyama et al	5	111	11	111	27.0%	0.43 [0.14, 1.28]
**Total (95% CI)**		**564**		**752**	**100.0%**	**0.80 [0.35, 1.86]**
Total events	41		55			

Heterogeneity: Tau² = 0.37; Chi² = 6.26, df = 2 (P = 0.04); I² = 68%
Test for overall effect: Z = 0.51 (P = 0.61)

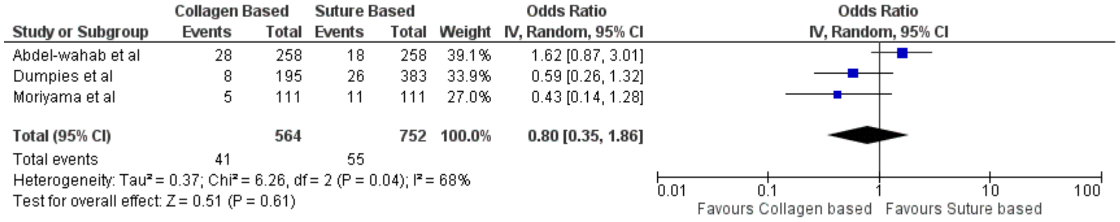

Supplementary Figure S2. Forest plot for secondary analysis of major and minor bleeding complications (excluding Prostar XL S-VCD), CI: confidence interval, IV: Instrumental variable.

Major Vascular Complications

Study or Subgroup	Collagen based Events	Collagen based Total	Suture based Events	Suture based Total	Weight	Odds Ratio IV, Random, 95% CI
Abdel-wahab et al	13	258	5	258	22.6%	2.68 [0.94, 7.64]
Biancari et al	10	107	14	115	25.8%	0.74 [0.32, 1.75]
Dumpies et al	4	195	25	383	22.2%	0.30 [0.10, 0.87]
Moriyama et al	8	111	9	111	23.5%	0.88 [0.33, 2.37]
Wiechen et al	2	102	0	104	5.9%	5.20 [0.25, 109.63]
**Total (95% CI)**		**773**		**971**	**100.0%**	**0.95 [0.42, 2.12]**
Total events	37		53			

Heterogeneity: Tau² = 0.46; Chi² = 9.71, df = 4 (P = 0.05); I² = 59%
Test for overall effect: Z = 0.13 (P = 0.89)

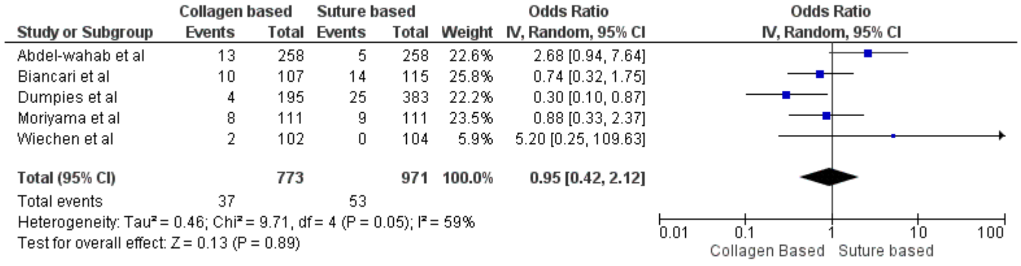

Minor Vascular Complications

Study or Subgroup	Collagen based Events	Collagen based Total	Suture based Events	Suture based Total	Weight	Odds Ratio IV, Random, 95% CI
Abdel-wahab et al	41	258	33	258	32.3%	1.29 [0.79, 2.11]
Biancari et al	4	107	3	115	6.5%	1.45 [0.32, 6.63]
Dumpies et al	17	195	48	383	27.3%	0.67 [0.37, 1.19]
Medranda et al	6	124	6	124	10.3%	1.00 [0.31, 3.19]
Moriyama et al	7	111	14	111	14.3%	0.47 [0.18, 1.20]
Wiechen et al	8	102	4	104	9.3%	2.13 [0.62, 7.30]
**Total (95% CI)**		**897**		**1095**	**100.0%**	**0.96 [0.64, 1.44]**
Total events	83		108			

Heterogeneity: Tau² = 0.07; Chi² = 6.98, df = 5 (P = 0.22); I² = 28%
Test for overall effect: Z = 0.21 (P = 0.84)

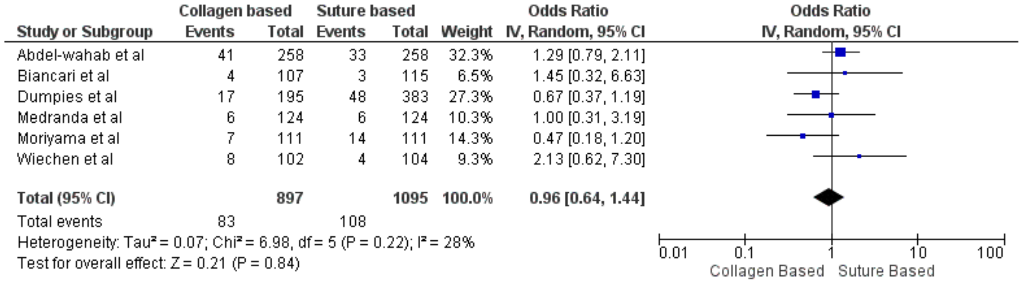

Supplementary Figure S3. Forest plot for secondary analysis of major and minor vascular complications (excluding Prostar XL S-VCD), CI: confidence interval, IV: Instrumental variable.

VCD Failure

Study or Subgroup	Collagen Based Events	Collagen Based Total	Suture Based Events	Suture Based Total	Weight	Odds Ratio IV, Random, 95% CI
Abdel-wahab et al	12	258	14	258	39.1%	0.85 [0.39, 1.88]
Biancari et al	4	107	8	115	16.2%	0.52 [0.15, 1.78]
Dumpies et al	7	195	12	383	27.2%	1.15 [0.45, 2.97]
Moriyama et al	5	111	7	111	17.6%	0.70 [0.22, 2.28]
**Total (95% CI)**		**671**		**867**	**100.0%**	**0.82 [0.50, 1.35]**
Total events	28		41			

Heterogeneity: Tau² = 0.00; Chi² = 1.10, df = 3 (P = 0.78); I² = 0%
Test for overall effect: Z = 0.77 (P = 0.44)

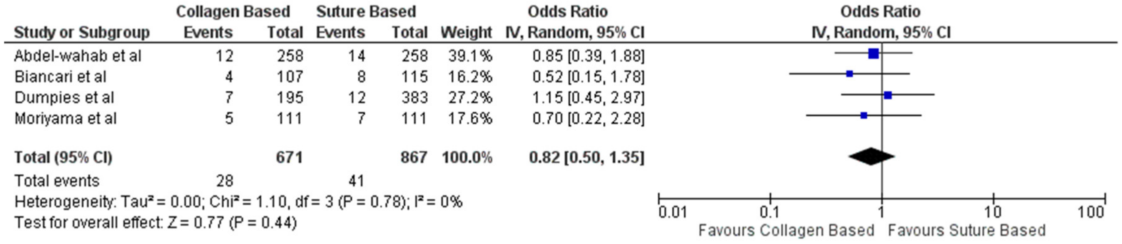

Stroke

Study or Subgroup	Collagen Based Events	Collagen Based Total	Suture Based Events	Suture Based Total	Weight	Odds Ratio IV, Random, 95% CI
Abdel-wahab et al	7	258	3	258	42.8%	2.37 [0.61, 9.27]
Biancari et al	0	107	1	115	7.7%	0.36 [0.01, 8.81]
Moriyama et al	5	111	5	111	49.5%	1.00 [0.28, 3.56]
**Total (95% CI)**		**476**		**484**	**100.0%**	**1.34 [0.55, 3.26]**
Total events	12		9			

Heterogeneity: Tau² = 0.00; Chi² = 1.53, df = 2 (P = 0.46); I² = 0%
Test for overall effect: Z = 0.64 (P = 0.52)

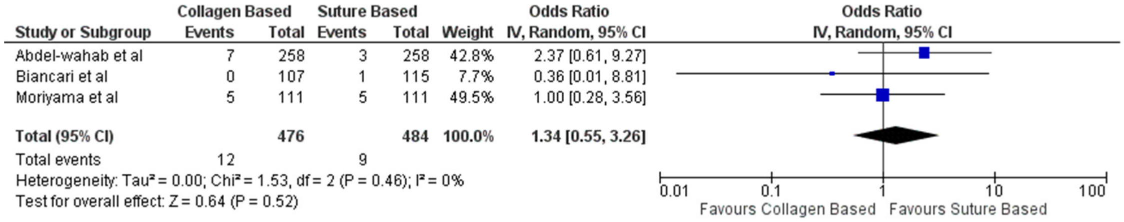

30-Day All Cause Mortality

Study or Subgroup	Collagen Based Events	Collagen Based Total	Suture Based Events	Suture Based Total	Weight	Odds Ratio IV, Random, 95% CI
Abdel-wahab et al	7	258	5	258	43.9%	1.41 [0.44, 4.51]
Biancari et al	0	107	2	115	10.1%	0.21 [0.01, 4.45]
Dumpies et al	3	195	6	383	35.0%	0.98 [0.24, 3.97]
Moriyama et al	0	111	5	111	11.0%	0.09 [0.00, 1.59]
**Total (95% CI)**		**671**		**867**	**100.0%**	**0.75 [0.27, 2.10]**
Total events	10		18			

Heterogeneity: Tau² = 0.27; Chi² = 3.94, df = 3 (P = 0.27); I² = 24%
Test for overall effect: Z = 0.54 (P = 0.59)

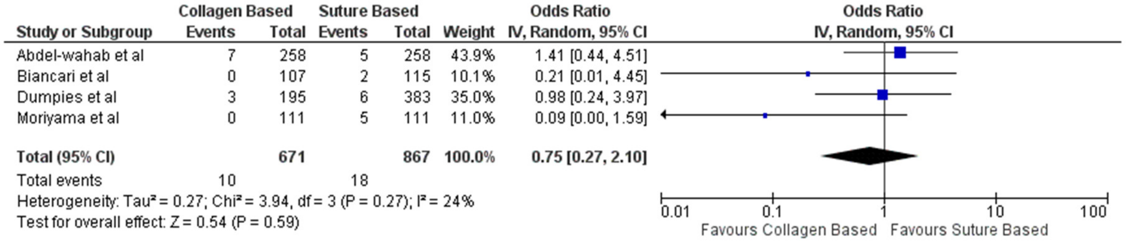

Supplementary Figure S4. Forest plot of for secondary analysis of secondary outcomes (excluding Prostar XL S-VCD): CI: Confidence interval, IV: Instrumental variable, VCD: Vascular closure device.

Supplementary Table S1. Assignment of variables to the outcomes.

Variable name	Outcome
O1	Major Bleeding Complications
O2	Minor Bleeding Complications
O3	Major Vascular Complications
O4	Minor Vascular Complications
O5	Stroke
O6	VCD failure
O7	30-day all-cause mortality

Supplementary Table S2. Risk of bias analysis for Medranda et al for primary and secondary outcomes.

Medranda et al							
	O1	O2	O3	O4	O5	O6	O7
Due to confounding	NA	NA	NA	Moderate	NA	NA	NA
Selection of participants into the study	NA	NA	NA	Low	NA	NA	NA
Classification of interventions	NA	NA	NA	Low	NA	NA	NA
Deviations from intended interventions	NA	NA	NA	Moderate	NA	NA	NA
Missing data	NA	NA	NA	Low	NA	NA	NA
Measurements of outcomes	NA	NA	NA	Low	NA	NA	NA

Selection of the reported result	NA	NA	NA	Low	NA	NA	NA
Overall bias	NA	NA	NA	Moderate	NA	NA	NA

Supplementary Table S3. Risk of bias analysis for Dumpies et al for primary and secondary outcomes.

Dumpies et al							
	O1	O2	O3	O4	O5	O6	O7
Due to confounding	Serious	Serious	Serious	Serious	NA	Serious	Serious
Selection of participants into the study	Low	Low	Low	Low	NA	Low	Low
Classification of interventions	Low	Low	Low	Low	NA	Low	Low

Deviations from intended interventions	Moderate	Moderate	Moderate	Moderate	NA	Moderate	Moderate
Missing data	Low	Low	Low	Low	NA	Low	Low
Measurements of outcomes	Low	Low	Low	Low	NA	Low	Low
Selection of the reported result	Low	Low	Low	Low	NA	Low	Low
Overall bias	Moderate	Moderate	Moderate	Moderate	NA	Moderate	Moderate

Supplementary Table S4. Risk of bias analysis for Noriaki et al for primary and secondary outcomes.

Noriaki et al							
	O1	O2	O3	O4	O5	O6	O7
Due to confounding	Moderate	Moderate	Moderate	Moderate	Moderate	Moderate	Moderate
Selection of participants into the study	Low	Low	Low	Low	Low	Low	Low
Classification of interventions	Low	Low	Low	Low	Low	Low	Low
Deviations from intended interventions	Moderate	Moderate	Moderate	Moderate	Moderate	Moderate	Moderate
Missing data	Low	Low	Low	Low	Low	Low	Low
Measurements of outcomes	Low	Low	Low	Low	Low	Low	Low

Selection of the reported result	Low	Low	Low	Low	Low	Low	Low
Overall bias	Moderate	Moderate	Moderate	Moderate	Moderate	Moderate	Moderate

Supplementary Table S5. Risk of bias analysis for Gheroge et al for primary and secondary outcomes.

Gheorge et al							
	O1	O2	O3	O4	O5	O6	O7
Due to confounding	Serious	Serious	Serious	Serious	N A	Serious	Serious
Selection of participants into the study	Low	Low	Low	Low	N A	Low	Low

Classification of interventions	Low	Low	Low	Low	NA	Low	Low
Deviations from intended interventions	Moderate	Moderate	Moderate	Moderate	NA	Moderate	Moderate
Missing data	Low	Low	Low	Low	NA	Low	Low
Measurements of outcomes	Low	Low	Low	Low	NA	Low	Low
Selection of the reported result	Low	Low	Low	Low	NA	Low	Low
Overall bias	Moderate	Moderate	Moderate	Moderate	NA	Moderate	Moderate

Supplementary Table S6. Risk of bias analysis for Hoffman et al for primary and secondary outcomes.

Hoffman et al							
	O1	O2	O3	O4	O5	O6	O7
Due to confounding	Serious	NA	NA	NA	NA	NA	NA
Selection of participants into the study	Low	NA	NA	NA	NA	NA	NA
Classification of interventions	Low	NA	NA	NA	NA	NA	NA
Deviations from intended interventions	Moderate	NA	NA	NA	NA	NA	NA
Missing data	Low	NA	NA	NA	NA	NA	NA
Measurements of outcomes	Low	NA	NA	NA	NA	NA	NA

Selection of the reported result	Low	NA	NA	NA	NA	NA	NA
Overall bias	Moderate	NA	NA	NA	NA	NA	NA

Supplementary Table S7. Risk of bias analysis for Biancari et al for primary and secondary outcomes.

Biancari et al							
	O1	O2	O3	O4	O5	O6	O7
Due to confounding	NA	NA	Serious	Serious	Serious	Serious	Serious
Selection of participants into the study	NA	NA	Low	Low	Low	Low	Low
Classification of interventions	NA	NA	Low	Low	Low	Low	Low

Deviations from intended interventions	NA	NA	Moderate	Moderate	Moderate	Moderate	moderate
Missing data	NA	NA	Low	Low	Low	Low	Low
Measurements of outcomes	NA	NA	Low	Low	Low	Low	Low
Selection of the reported result	NA	NA	Low	Low	Low	Low	Low
Overall bias	NA	NA	Moderate	Moderate	Moderate	Moderate	Moderate

Supplementary Table S8. Risk of bias analysis for DePalma et al for primary and secondary outcomes.

DePalma et al							
	O1	O2	O3	O4	O5	O6	O7
Due to confounding	Serious	Serious	Serious	Serious	Serious	NA	Serious
Selection of participants into the study	Low	Low	Low	Low	Low	NA	Low
Classification of interventions	Low	Low	Low	Low	Low	NA	Low
Deviations from intended interventions	Moderate	Moderate	Moderate	Moderate	Moderate	NA	Moderate
Missing data	Low	Low	Low	Low	Low	NA	Low

Measurements of outcomes	Low	Low	Low	Low	Low	NA	Low
Selection of the reported result	Low	Low	Low	Low	Low	NA	Low
Overall bias	Moderate	Moderate	Moderate	Moderate	Moderate	NA	Moderate

Supplementary Table S9. Risk of bias analysis for Weichen et al for primary and secondary outcomes.

Wiechen et al							
	O1	O2	O3	O4	O5	O6	O7
Randomization	NA	NA	Low	Low	NA	NA	NA
Selection of participants into the study	NA	NA	Low	Low	NA	NA	NA
Deviations from intended interventions	NA	NA	Low	Low	NA	NA	NA

Missing outcome data	NA	NA	Low	Low	NA	NA	NA
Measurements of outcomes	NA	NA	Low	Low	NA	NA	NA
Selection of the reported result	NA	NA	Low	Low	NA	NA	NA
Overall bias	NA	NA	Low	Low	NA	NA	NA

Supplementary Table S10. Risk of bias analysis for Abdel-Wahab et al for primary and secondary outcomes.

Abdel-Wahab et al							
	O1	O2	O3	O4	O5	O6	O7
Randomization	Low	Low	Low	Low	Low	Low	Low
Selection of participants into the study	Low	Low	Low	Low	Low	Low	Low
Deviations from intended interventions	Low	Low	Low	Low	Low	Low	Low
Missing outcome data	Low	Low	Low	Low	Low	Low	Low
Measurements of outcomes	Low	Low	Low	Low	Low	Low	Low
Selection of the reported result	Low	Low	Low	Low	Low	Low	Low
Overall bias	Low	Low	Low	Low	Low	Low	Low

Legends:

Supplementary Figure S1: Forest plot for analysis of major and minor bleeding complications (adjusted to decrease heterogeneity), CI: confidence interval, IV: Instrumental variable

Supplementary Figure S2: Forest plot for secondary analysis of major and minor bleeding complications (excluding Prostar XL S-VCD), CI: confidence interval, IV: Instrumental variable

Supplementary Figure S3: Forest plot for secondary analysis of major and minor vascular complications (excluding Prostar XL S-VCD), CI: confidence interval, IV: Instrumental variable

Supplementary Figure S4: Forest plot of for secondary analysis of secondary outcomes (excluding Prostar XL S-VCD): CI: Confidence interval, IV: Instrumental variable, VCD: Vascular closure device.

Supplementary table S1: Assignment of variables to the outcomes.

Supplementary table S2: Risk of bias analysis for Medranda et al for primary and secondary outcomes:

Supplementary table S3: Risk of bias analysis for Dumpies et al for primary and secondary outcomes:

Supplementary table S4: Risk of bias analysis for Noriaki et al for primary and secondary outcomes:

Supplementary table S5: Risk of bias analysis for Gheroge et al for primary and secondary outcomes:

Supplementary table S6: Risk of bias analysis for Hoffman et al for primary and secondary outcomes:

Supplementary table S7: Risk of bias analysis for Biancari et al for primary and secondary outcomes:

Supplementary table S8: Risk of bias analysis for DePalma et al for primary and secondary outcomes:

Supplementary table S9: Risk of bias analysis for Weichen et al for primary and secondary outcomes:

Supplementary table S10: Risk of bias analysis for Abdel-Wahab et al for primary and secondary outcomes:
